# Evaluation of inner ear damage by mastoid drilling with measurement of serum prestin (SLC26A5) levels

**DOI:** 10.1016/j.bjorl.2023.101380

**Published:** 2023-12-19

**Authors:** Ayca Baskadem Yilmazer, Onur Tanrısever, Maide Hacer Alagoz, Rasim Yilmazer, Ayse Enise Goker, Belgin Tutar, Yavuz Uyar

**Affiliations:** a Saglik Bilimleri University, Prof. Dr. Cemil Tascioglu Hospital, Department of Otorhinolaryngology, Head and Neck Surgery, Istanbul, Turkey; b Saglik Bilimleri University, Prof. Dr. Cemil Tascioglu Hospital, Department of Biochemistry, Istanbul, Turkey; c Saglik Bilimleri University, Dr. Lutfi Kirdar City Hospital, Department of Otorhinolaryngology, Head and Neck Surgery, Istanbul, Turkey

**Keywords:** Prestin, SLC26A5, Mastoidectomy, Inner ear, Drill

## Abstract

**Objective:**

The objective of this study is to demonstrate any inner ear injury caused by drilling in mastoid surgery with prestin, outer hair cell motor protein specific to the cochlea.

**Methods:**

The patients with chronic otitis media requiring mastoidectomy (*n* = 21) and myringoplasty (*n* = 21) were included. Serum prestin level obtained from blood samples was measured before surgery and on postoperative days 0, 3, and 7 using Human Prestin (SLC26A5) ELISA Kit. All patients underwent the Pure Tone Audiometry (PTA) test before surgery and on the postoperative 7th day. The drilling time was also recorded for all patients who underwent mastoidectomy.

**Results:**

In both mastoidectomy and myringoplasty groups, the postoperative serum prestin levels increased on days 0 and 7 (*p*_day-0_ = 0.002, *p*_day-7_ = 0.001 and *p*_day-0_ = 0.005, *p*_day-7_ = 0.001, respectively). There was no significant difference in the serum prestin levels between the two groups, postoperatively. The PTA thresholds at day 7 did not change in either group. A significant decline at 2000 Hz of bone conduction hearing threshold in both groups and a decline at 4000 Hz in the myringoplasty group were found. There was no correlation between the drilling time and the increase of prestin levels in the postoperative day 0, 3, and 7.

**Conclusion:**

Our results showed that mastoid drilling is not related to a significant inner ear injury. Although the myringoplasty group was not exposed to drill trauma, there was a similar increase in serum prestin levels as the mastoidectomy group. Also, a significant decline at 2000 Hz of bone conduction hearing threshold in both groups and a decline at 4000 Hz in the myringoplasty group were found. These findings suggest that suction and ossicular manipulation trauma can lead to an increase in serum prestin levels and postoperative temporary or permanent SNHL at 2000 and 4000 Hz.

**Level of evidence:**

Level-4.

## Highlights

• Mastoid drilling is not related to a significant inner ear injury.
• Suction and ossicular manipulation trauma can increase serum prestin levels.
• Suction trauma can cause sensorineural hearing loss at 2000 and 4000 Hz.
• Ossicular manipulation trauma can cause sensorineural hearing loss at 2000 and 4000 Hz.

## Introduction

During all otology surgeries, such as myringoplasty, mastoidectomy, stapedotomy, and cochlear implantation, the first aim is to preserve the residual hearing of the patients. Besides this, Sensorineural Hearing Loss (SNHL) is an undesirable complication for both surgeons and patients. This can be a result of the pathology in the ear, types of equipment such as drills, suctions, lasers used in surgery, or iatrogenic conditions. It is difficult to pinpoint a single cause as various factors might be responsible for this complication.

SNHL is seen in 1.2%–4.5% of patients after chronic otitis media surgeries.^1^, ^2^ One of the possible causes is the drill usage in the surgeries. A drill can cause direct contact with the ossicles, trauma to the membranous labyrinth, or noise trauma to the inner ear.^3^ However, the effect of drilling on hearing loss is still a controversial point in the literature.^4^, ^5^

In many studies, the effect of drilling on hearing has been shown with hearing tests such as Pure Tone Audiometry (PTA), high-frequency electrostimulation audiometry, and Otoacoustic Emissions (OAEs).^4^, ^6^, ^7^ However, to evaluate the effect of the drilling on hearing, a blood test that is objective, feasible, reliable, and specific to the cochlea would be helpful.

Prestin (SLC26A5) is a motor protein located in the lateral membrane of the Outer Hair Cells (OHCs), and it is specific to the cochlea.^8^ Because it can pass through the blood-labyrinthine barrier and interfere with blood circulation, the serum concentration of prestin can be measured. In case of any damage to the cochlea, OHCs undergo apoptosis and prestin release into the bloodstream occurs from the phagocytized OHCs. Subsequently, the prestin concentration increases in the blood and can predict any damage to the cochlea. Therefore, Parham K. hypothesized that prestin protein may be used as a serum biomarker to detect inner ear injuries causing hearing loss.^9^ Serum prestin levels were measured in many animal studies demonstrating the cochlear injury related to ototoxicity or noise trauma.^10^, ^11^, ^12^ Several human studies have investigated the prestin levels in patients with different kinds of hearing loss such as noise-induced, sudden, or SNHL.^13^, ^14^, ^15^, ^16^, ^17^

In this study, we aimed to investigate the effect of drilling on the inner ear with the human serum prestin levels and pure tone audiometry in patients who underwent mastoidectomy surgeries.

## Methods

This was a prospective clinical study, and it was performed in a tertiary referral center between June 2020 and February 2021. We have performed canal wall-up mastoidectomy in patients with a dry tympanic membrane perforation and mastoid air cell opacification on Computed Tomography (CT) imaging. Patients with a diagnosis of cholesteatoma underwent canal wall-down mastoidectomy. Myringoplasty was performed in patients with a dry central tympanic membrane perforation and normal pneumatization in mastoid air cells. The patients undergoing canal wall-up mastoidectomy to explore the mastoid to ensure that there is no disease and patients undergoing canal wall-down mastoidectomy were assigned to the study (mastoidectomy) group, and the patients undergoing myringoplasty surgery were assigned to the control (myringoplasty) group. All procedures were performed by the same surgeon (ABY). Our inclusion criteria were patients with pure conductive type hearing loss, age 18–65 years old, Chronic Otitis Media (COM) with or without cholesteatoma, no otorrhea for ≥3 months prior to enrollment, and clear middle ear mucosa upon microscopic examination in the operating room. On the other hand, patients with SNHL or a history of otologic surgery, and any patients with comorbidities like diabetes mellitus, thyroid dysfunction, hyperlipidemia, and hypertension were excluded. Patients requiring ossicular chain reconstruction were scheduled for a second-look surgery.

The total study population was determined as 46 with a power analysis program by taking effect size *f* = 0.25, α = 0.05, power (1-β) = 0.90.

All patients underwent the PTA test before surgery and on day 7 after surgery. The PTA test was calculated using four tones at 500, 1000, 2000, and 4000 Hz. The pre- and post-operative audiograms were performed in a soundproof audiometry room by the same audiologist.

To measure prestin (SLC26A5) level in serum, 3 mL of blood were acquired from all of the patients before surgery and on days 0, 3, and 7 after surgery. Serum obtained via centrifugation at 2000 rpm for 10 min was frozen and stored at −80 °C. SLC26A5 was measured by using a Human Prestin (SLC26A5) Enzyme-linked Immunosorbent Assay (ELISA) Kit (SunRedBio Technology Co., Ltd, Shanghai, China) according to the manufacturer’s instructions.

The drill speed was between 20 and 60,000 rpm, and the round stainless-steel cutting and diamond burrs were between 1 and 4 mm in diameter. In all surgeries, the duration of the drilling was recorded.

To evaluate the data, descriptive statistical methods (mean, median, standard deviation “SD”, frequency, percentage, minimum, and maximum) were used. The normal distribution of quantitative variables was analyzed with the Shapiro-Wilks test and graphics. In the comparison of two groups with normally distributed quantitative variables, the Student’s *t*-test was used; but with distributions deviated from normal, the Mann-Whitney *U* test was used. The Friedman test was used for quantitative distributions that deviated from normal, and the Wilcoxon signed-rank test corrected with Bonferroni was used in analyzing two paired comparisons. The dependent *t*-test was used for normally distributed quantitative variables at the internal comparison of each group. For the comparison of qualitative variables, Pearson’s Chi-Squared test was used. To analyze the relationship between the quantitative variables, a Spearman correlation analysis was used. The statistical significance was accepted as *p* <  0.05.

This study was reviewed and approved by the Ethics Committee of Clinical Studies of Institutional Review Board and designed in accordance with the Helsinki Declaration. A written informed consent form was obtained from each participant.

## Results

Since we put 4 samples for each participant, and 1 blank and 5 controls for full functionality of all assay components, we used two packages of Human Prestin ELISA Kit-96 tests and we could have studied a total of 44 patients. However, at the end of the study, the specimens at postop day 7 of two patients, one in the mastoidectomy group and the other in the myringoplasty group were deficient. Therefore, we had to exclude those patients. Finally, 21 patients who underwent mastoidectomy and 21 patients who underwent myringoplasty were included in two groups. In the mastoidectomy group, there were 12 females (57.1%) and 9 males (42.9%). The average age was 37.48 ± 11.29 years. Mastoidectomy was performed in the right ear of 13 (61.9%) and left ear of 8 (38.1%) patients. Four patients had canal wall-down mastoidectomy and 17 patients had canal wall-up mastoidectomy. In the myringoplasty group, there were 11 females (52.4%) and 10 males (47.6%). The average age was 34.05 ± 10.85 years. Myringoplasty was performed in the right ear of 11 (52.4%) and left ear of 10 (47.6%) patients. Three patients who had canal wall-down mastoidectomy required ossicular chain reconstruction in a second-look surgery. In descriptive statistics, there was no statistically significant difference between the two groups in terms of age, gender, and side of the ear with the disease.

In both groups, serum prestin level was measured in all patients at the preoperative time, and postoperative days 0, 3, and 7. In the mastoidectomy group, preoperative prestin levels ranged from 296‒2489 pg/mL (median = 438.71 pg/mL). The average prestin level was 645.53 ± 516.45 pg/mL (mean ± SD) preoperatively. There was a significant increase in serum prestin levels after mastoidectomy on Postoperative day 0 (*p* =  0.002) and 7 (*p* =  0.001) which were 744.9 ± 499.84 pg/mL and 799.14 ± 607.03 pg/mL, respectively. On day 3 after surgery, serum prestin level decreased to 686.58 ± 480.35 pg/mL but it was still higher than the preoperative level.

In the myringoplasty (control) group, preoperative prestin levels ranged from 297.2‒1172.1 pg/mL (median = 457 pg/mL). The preoperative average prestin level was 555.7 ± 259.4 pg/mL. There was a significant increase in serum prestin levels after myringoplasty on Postoperative day 0 (*p* =  0.005) and 7 (*p* =  0.001) which were 670.74 ± 353.32 and 766.04 ± 398.66 pg/mL, respectively. On day 3 after surgery, serum prestin level decreased to 620.49 ± 360.32 pg/mL but it was still higher than the preoperative level.

**Figure 1:**
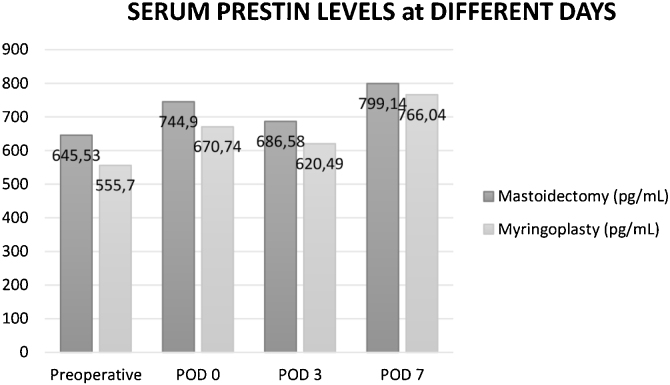
Comparison of serum prestin levels of two groups at different days.

There was no statistically significant difference between the prestin levels of the two groups when they were compared on each day, both preoperatively and postoperatively. Moreover, when the increment in prestin levels between the preoperative and postoperative days was compared between the two groups, there was no statistically significant difference (Fig. 1).

In the preoperative period and postoperative day 7, PTA (500–4000 Hz) was performed on all patients. In the mastoidectomy group, at the preoperative period, the average bone conduction hearing threshold was significantly higher (worse) than that in the myringoplasty group (16.19 ± 6.88 dB; 10.24 ± 5.28 dB, respectively) (*p* =  0.003; *p* < 0.01). On postoperative day 7, the average bone conduction hearing threshold was significantly higher (worse) in the mastoidectomy group than that in the myringoplasty group (18.33 ± 6.81 dB; 13.05 ± 9.24 dB, respectively) (*p* =  0.041; *p* <  0.05).

**Figure 2:**
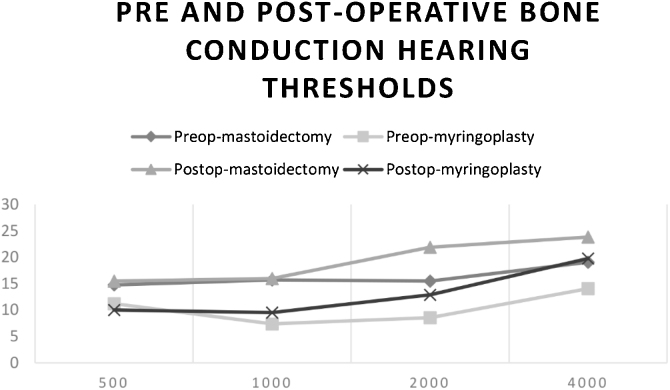
Comparison of the bone conduction hearing thresholds of both groups at pre and postoperative periods.

There was a significant decline at 2000 Hz of bone conduction hearing threshold in mastoidectomy and myringoplasty groups which were 6.43 ± 11.08 dB (*p* =  0.018) and 4.29 ± 8.26 dB (*p* =  0.03), respectively. There was also a significant decline at 4000 Hz of bone conduction hearing threshold in the myringoplasty group which was 5.71 ± 11.97 dB (*p* =  0.048). In each group, there was no statistically significant difference between the preoperative and postoperative day 7 average bone conduction hearing thresholds (Fig. 2).

In the mastoidectomy group, the average drilling time was 42.05 ± 14.3 min (min. 20; max. 74; median 42). There was no correlation between the drilling time and the increase of prestin levels in the postoperative day 0, 3, and 7 (*p* =  0.137; *p* =  0.762; *p* =  0.107; respectively).

## Discussion

Hearing loss because of the drilling during ear surgeries is still controversial in the literature. Several studies showed that drilling generates high noise levels between 103 and 139 dB.^7^, ^8^, ^19^ In a clinical study, Palva and Sorri performed simple or modified radical mastoidectomy in 55 patients and followed the hearing of the contralateral ear by PTA to obtain information about the acoustic trauma caused by drilling.^20^ They reported 12 patients with SNHL of at least 20 dB in the contralateral ear. This hearing loss was persistent in 6 cases. They also concluded that the number of patients with hearing loss had increased with increasing operation time (>3 h). Urquhart et al. obtained the PTA results within 24 h of mastoidectomy surgeries and declared that there was no difference between the pre-and postoperative bone conduction hearing results.^4^ In the present study, we measured the PTA on day 7 and found that there was no difference between the pre-and postoperative average bone conduction hearing thresholds in the mastoidectomy group that underwent the drilling.

The duration of drilling is an important factor that can be managed to minimize the noise trauma to the inner ear. Goyal et al. investigated the effect of mastoid drilling on the contralateral ear of 30 patients. They reported no significant change in bone conduction hearing thresholds. The mean drilling time was 53.93 min.^21^ Similar to that study, in our study, the mean drilling time was 42.05 min. We compared the drilling time and prestin levels in the mastoidectomy group and found that the postoperatively increased serum prestin levels were not related to the duration of drilling.

The prestin protein was first described by Zheng et al. in 2000.^8^ Later, Parham and Dyhrfjeld-Johnsen claimed that changes in serum prestin levels were detectable in the early stage of inner ear trauma before any shifts in audiometric thresholds.^10^ In another study, Parham et al. worked in noise trauma-exposed rats and found the average baseline prestin levels to be 177.9 ± 4.3 pg/mL.^22^ In a study on ototoxicity, baseline prestin levels in rats were 377.0 ± 135.3 pg/mL.^12^ According to the theory of Parham, serum prestin levels in control patients result in normal homeostatic turnover in the membrane of healthy outer hair cells. Shortly after outer hair cell damage, prestin concentration will rise and reach a peak sometime within 1 week. When OHCs die, with time, there is less release of prestin into circulation, and prestin levels are lower than baseline 14 days after exposure to noise.^11^, ^22^ Parker et al. studied serum prestin levels in healthy adults with normal hearing and provided initial normative data that may help interpret results from individuals with SNHL. The mean serum prestin level was 250.20 pg/mL, with a standard error of measurement of 7.28 pg/mL in young adults between 18 and 24 years old. They have also found in another study that serum prestin levels negatively correlate with age, with young adults having higher levels of circulating serum in the blood than older adults. The mean prestin level was 231.72 pg/mL.^23^ In addition, serum prestin levels declined with increasing levels of hearing loss.^15^ Sun et al. investigated the role of prestin levels in the patients with idiopathic sudden SNHL and found the baseline prestin levels in the control patients as 840.24 ± 496.22 pg/mL.^14^ In our study, in the mastoidectomy group, the baseline serum prestin level (preoperatively) was 645.53 ± 516.45 pg/mL and in the myringoplasty group, it was 555.7 ± 259.4 pg/mL. The higher baseline serum prestin levels in our patients might be attributed to many factors. Intermittent infections and noise trauma without the protective effect of the tympanic membrane in patients with tympanic membrane perforation could increase the damaged OHCs and serum prestin levels. The average ages of the mastoidectomy and myringoplasty groups were 37 and 34 years old, respectively. As the younger patients tend to have higher serum prestin levels,^23^ this factor could contribute to the high levels of serum prestin in our study.

In case of any injury to OHCs, prestin increases in the serum, and after a while decreases.^10^ Liba et al. studied guinea pigs and mice after giving them cisplatin, and they measured prestin levels on days 1, 3, 7, and 14. They showed that the peak prestin levels were at day 3 in guinea pigs and day 7 in mice.^11^ Parham et al. studied serum prestin levels in rats on days 1 and 14 after noise trauma and they declared that after noise trauma, prestin levels increased at day 1 and decreased at day fourteen.^22^ Sun et al. studied prestin levels in human beings. They reported that the average prestin level in patients with sudden hearing loss was significantly higher than that of control patients. They measured the serum prestin levels of patients after sudden hearing loss at day 0 before treatment and day 7 after treatment and the mean values were 1955.98 ± 2501.48 pg/mL and 1653.26 ± 1967.60 pg/mL, respectively. They found no correlation between the serum prestin levels before and after the treatment.^14^ We observed that there was an increase in prestin levels at days 0, 3, and 7 in comparison to one on the preoperative day in both groups. In our study, the peak period of the serum prestin levels was observed on day 7 in both mastoidectomy and myringoplasty groups and measured 799.14 ± 607.03 and 766.04 ± 398.66 pg/mL, respectively. Although the myringoplasty group was not exposed to drill trauma, there was a similar increase in serum prestin levels as the mastoidectomy group. We thought that the increase in prestin levels in the myringoplasty group might be due to suction trauma and ossicular manipulation. Yin et al. reported that noise levels generated by various types of suctions ranged from 100 to 129 dB sound pressure level.^18^ Kazikdas et al. investigated SNHL after ossicular manipulation and drill-generated acoustic trauma in type 1 tympanoplasty with and without mastoidectomy in a series of 51 patients. They reported that, in the tympanoplasty group, a significant SNHL, primarily at 2 kHz, was seen in 6 patients (23%) at 24 h, but at 6 months there was no depression of bone-conduction thresholds. They also reported that, in the mastoidectomy group, a significant SNHL, primarily at 2 and 4 kHz, occurred in 12 patients (48%) at 24 h, and bone-conduction deterioration was still present in 4 patients (16%) 6 months after surgery.^24^ Similarly, in our study, we found a significant decline at 2000 Hz of bone conduction hearing threshold in the mastoidectomy and myringoplasty groups and a decline at 4000 Hz in the myringoplasty group. The increase of prestin levels in the myringoplasty group can also be due to the intact ossicular chain which makes those patients more vulnerable to suction and ossicular manipulation trauma. Because some of the patients in the mastoidectomy group had disrupted the ossicular chain which can protect them from noise trauma. Tos et al. reported that the incidence of postoperative SNHL was higher in ears with an intact ossicular chain than a disrupted chain.^2^ Therefore, these findings suggest that suction and ossicular manipulation trauma can lead to an increase in serum prestin levels and postoperative temporary or permanent SNHL at 2 and 4 kHz.

There are some limitations of our study. The sample size was small. Further studies can be performed with larger sample sizes. In order to more accurately obtain the peak and decreasing periods of serum prestin levels and evaluate the effects of drill and noise trauma on hearing, serum prestin levels and PTA could be measured on days 14, 30, and 180. Another limitation that must be considered is that prestin is expressed in the heart.^25^ However, Naples et al. investigated the possibility of prestin being expressed in other tissues and found that expression in the heart was much lower than levels found in the bloodstream. They thought that most of the prestin in circulation was likely from the inner ear.^26^ However, we can not rule out a cardiac contribution.

## Conclusion

Our results showed that mastoid drilling is not related to a significant inner ear injury. Although the myringoplasty group was not exposed to drill trauma, there was a similar increase in serum prestin levels as the mastoidectomy group. Also, a significant decline at 2000 Hz of bone conduction hearing threshold in both groups and a decline at 4000 Hz in the myringoplasty group were found. These findings suggest that suction and ossicular manipulation trauma can lead to an increase in serum prestin levels and postoperative temporary or permanent SNHL at 2000 and 4000 Hz. Further studies are needed with larger sample sizes and long-term follow-up to evaluate the effects of drill and noise trauma on hearing after ear surgeries.

## Authors' contributions

Ayca Baskadem Yilmazer: Conceptualization, Methodology, Writing-original draft. Onur Tanrisever: Data analysis and Resources. Maide Hacer Alagoz: Data analysis and Resources. Rasim Yilmazer: Revising the study and writing. Ayse Enise Goker: Data analysis and interpretation. Belgin Tutar: Data analysis and interpretation. Yavuz Uyar: Final approval and Supervision. All authors contributed to the writing of the final manuscript.

## Funding

This research did not receive any specific grant from funding agencies in the public, commercial, or not-for-profit sectors. It is funded by the authors.

## Conflicts of interest

The authors declare no conflicts of interest.
